# Feedforward Pre‐Activation of Lateral Ankle Muscles Is Insufficient Against Excessive Ankle Inversion During Perturbation in Individuals With Chronic Ankle Instability

**DOI:** 10.1002/jfa2.70232

**Published:** 2026-10-12

**Authors:** Wenqi Ran, Qipeng Song, Wei Yin

**Affiliations:** ^1^ College of Sports and Health Shandong Sport University Jinan China; ^2^ China National Basketball Academy Shandong Sport University Jinan China

**Keywords:** electromyography, inversion perturbations, kinematics, neuromuscular control, peroneus longus

## Abstract

**Objective:**

This study aimed to compare ankle kinematics and muscle activation between individuals with and without chronic ankle instability (CAI), both prior to and following landing on an inversion trapdoor during walking, with a particular focus on the feedforward pre‐activation of lateral ankle muscles and the consequent ankle kinematics post‐perturbation.

**Methods:**

Forty participants were enrolled, including 20 with CAI and 20 age‐ and sex‐matched non‐CAI controls. They walked at their own pace on a custom‐designed walking platform equipped with a trapdoor programmed to trigger a 24° inversion perturbation, simulating lateral ankle sprain conditions, across three unanticipated trials. Their lower limb muscle electromyography and ankle kinematics were synchronously collected 200 ms pre‐ and 200 ms post‐perturbation. Continuous between‐group data were compared by independent sample t‐tests.

**Results:**

CAI individuals showed higher pre‐activation in the peroneus longus (−200.0 to −196.4 ms, mean _diff_: −11.38%, *p* = 0.049; −54.6 to −11.0 ms, mean _diff_: −10.60%, *p* = 0.011) and the lateral gastrocnemius (−200.0 to −175.4 ms, mean _diff_: −7.62%, *p* = 0.029) prior to perturbation, and lower activation in the peroneus longus at 72.1 to 98.0 ms (mean _diff_: 17.46%, *p* = 0.029) post‐perturbation. They also showed greater ankle inversion in four clusters post‐perturbation (0 to 111.0 ms, mean _diff_: −4.85°, *p* < 0.001; 117.0 to 128.0 ms, mean _diff_: −1.83°, *p* = 0.020; 142.0 to 145.0 ms, mean _diff_: −1.30°, *p* = 0.047; 154.0 to 165.0 ms, mean _diff_: −1.05°, *p* = 0.019).

**Conclusion:**

CAI individuals exhibit higher feedforward pre‐activation in lateral ankle muscles, which may be insufficient against excessive ankle inversion after landing on a trapdoor during walking.

## 1 Introduction

Ankle sprains are among the most common musculoskeletal injuries of the lower extremity, with lateral ankle sprains (LAS) accounting for approximately 80%–90% of all ankle injury cases [1]. Following an initial LAS, up to 40% of individuals develop chronic ankle instability (CAI) [2], characterized by recurrent episodes of the ankle ‘giving way’, persistent pain, and functional limitations [3].

Dynamic ankle stability relies on the integration of feedforward (preparatory) and feedback (reactive) muscle activation [4]. However, due to the rapid onset of acute inversion sprains after initial contact with the ground and the physiological constraints of nerve conduction, feedback mechanisms are often too slow to prevent ligamentous loading [5]. Biomechanical data indicate that injurious ankle inversion typically occurs within 60–80 ms following initial contact [6]. In contrast, the reaction time required for the peroneus muscles to generate ankle eversion torque often exceeds this critical window of 80 ms. This physiological time lag implies that feedback mechanisms may be ineffective in preventing ligamentous injuries. Furthermore, this temporal deficit is exacerbated in individuals with CAI due to delayed reactions resulting from proprioceptive deficits [7].

To compensate for feedback delays, feedforward muscle activation prior to perturbation may serve as a critical mechanism for maintaining dynamic ankle stability [8]. However, research focusing on this strategy remains relatively scarce, and existing conclusions regarding feedforward muscle activation in individuals with CAI are inconsistent. Some studies have observed lower pre‐activation in the PL in those with CAI during drop‐landing [9, 10], while others have detected higher pre‐activation in the PL during level walking, suggesting a task‐specific ‘compensatory enhancement’ strategy [11, 12]. Furthermore, current literature is limited to isolated descriptions of muscle activation, lacking in‐depth coupled analysis between muscle activation and ankle kinematics. Consequently, it remains unclear whether the alterations in muscle activation during the feedforward phase, manifesting as either inhibition or enhancement, effectively translate into functional adjustments in ankle kinematics.

Sudden ankle inversion triggered by external perturbations during walking represents a common scenario of recurrent LASs in individuals with CAI [13]. However, existing literature has been largely confined to routine tasks such as static single‐leg stance or level walking [11]. While these studies provide valuable insights, they fail to adequately replicate the scenarios of LASs due to the lack of sudden ankle inversion. Compared to static or steady‐state conditions, responding to sudden inversion perturbations imposes complex demands on the regulatory capacity of the neuromuscular system. This task‐dependent interpretation is supported by a recent review, which suggested that high‐demand tasks, such as unexpected perturbations, may expose deficits in rapid protective responses that are less apparent during steady‐state tasks. Although a limited number of studies have utilized drop‐landing tasks on tilted platforms to simulate perturbation scenarios [14, 15], these protocols often involve anticipated responses that do not fully capture the unpredictable nature of real‐world sprains. Consequently, research simultaneously examining ankle kinematics and muscle activation during unexpected sudden inversion in continuous walking remains scarce, despite this scenario more closely approximating actual injury mechanisms. Furthermore, traditional discrete analyses often mask transient neuromuscular fluctuations, leaving the continuous temporal mapping of these dynamic responses largely unexplored.

Therefore, this study aims to continuously compare ankle kinematics and lower leg muscle activation between individuals with CAI and their counterparts during simulated walking inversion perturbations using Statistical Parametric Mapping (SPM). We specifically examined the muscle activation prior to perturbation to determine whether individuals with CAI utilize distinct feedforward strategies, and to investigate the subsequent ankle kinematics after the perturbation. It is hypothesized that, compared to individuals without CAI, those with CAI will exhibit higher pre‐activation of the lateral ankle muscles, such as the PL and LG, prior to perturbation (Hypothesis #1). This increased pre‐activation is expected to counteract the ankle inversion tendency observed in CAI individuals, resulting in no significant between‐group differences in ankle inversion angles during either the pre‐perturbation (Hypothesis #2) or post‐perturbation phases (Hypothesis #3).

## 2 Materials and Methods

### 2.1 Participants

The sample size was estimated based on the ankle inversion angle data from our pilot study, where a significant difference between individuals with CAI (14.28° ± 3.42°) and their counterparts without CAI (10.83° ± 3.00°) was detected with a large effect size (Cohen's *d* = 1.07). An a priori power analysis was performed using G*Power software (Version 3.1, Heinrich Heine University, Düsseldorf, Germany), indicating that a minimum of 15 participants per group was required to achieve a statistical power of 0.80 at an alpha level of 0.05. To allow for potential attrition, the target sample size was set at 40 participants, with 20 participants in each group.

A total of 63 volunteers from a local university responded to recruitment flyers and were assessed for eligibility. Of these, 23 volunteers were excluded: 10 did not meet the inclusion criteria, including Cumberland Ankle Instability Tool (CAIT) scores outside the required range (*n* = 10); 7 met the exclusion criteria, including a history of lower limb surgery or fracture (*n* = 4) and acute injury within the previous 3 months (*n* = 3); and 6 were excluded for other reasons, including declining to participate (*n* = 3) and bilateral CAI (*n* = 3). Therefore, 40 eligible participants were enrolled, including 20 individuals with CAI (9 females and 11 males, aged 20.3 ± 1.8 years, with a height of 172.4 ± 8.7 cm and a weight of 67.4 ± 11.0 kg; CAIT score = 15.5 ± 5.2) and 20 individuals without CAI (9 females and 11 males, aged 20.9 ± 1.6 years, with a height of 171.2 ± 5.4 cm and a weight of 66.6 ± 11.4 kg; CAIT score = 27.1 ± 1.3). No participants withdrew or were excluded during data collection or data processing; data from all 40 enrolled participants were included in the final analysis. Detailed demographic and anthropometric data are presented in Table 1. The inclusion criteria for individuals with CAI strictly followed the criteria established by the International Ankle Consortium [16], which included: (a) Aged 18–24 years, inclusive of both sexes; (b) At least two prior lateral ankle sprains, with at least one in the past 12 months that interrupted physical activity for at least one day; (c) At least two episodes of ankle ‘giving way’ within the 6 months preceding enrollment; (d) A CAIT score of less than 24; and (e) Physical activity ≥ 3 days/week for 90 min within the past 3 months. The inclusion criteria for individuals without CAI were: (a) No history of previous LAS; (b) Matched to the CAI participants in terms of sex, age (± 3 years), height (± 5 cm), and weight (± 5 kg); (c) A CAIT score of 28 or higher; and (d) Physical activity ≥ 3 days/week for 90 min within the past 3 months. The exclusion criteria for both groups were: (a) A history of lower extremity fracture or surgery; (b) An acute lower extremity injury in the preceding 3 months; (c) Diagnosed neurological, vestibular, or metabolic (e.g., diabetes) disorders; or (d) Bilateral CAI. There were no significant differences in age, height, or body mass between the groups (*p* > 0.05). All participants provided written informed consent, and the study was approved by the Institutional Review Board of Shandong Sport University (No. 2025053) and was in accordance with the Declaration of Helsinki.

**TABLE 1:** Significant clusters for ankle kinematics and muscle activation.

Variable	Cluster location (ms)	Duration (ms)	*p*	t*	Mean _diff_	Cohen's *d*_max_
Ankle kinematics						
Ankle inversion angle	0 to 111.0	112	< 0.001	−15.62	−4.85°	4.95
	117.0 to 128.0	11	0.020	−4.190	−1.83°	1.32
	142.0 to 145.0	3	0.047	−3.568	−1.30°	1.13
	154.0 to 165.0	11	0.019	−3.689	−1.05°	1.17
Muscle activation						
Peroneus Longus	−200.0 to −196.4	4	0.049	−3.13	−11.38%	0.98
	−54.6 to −11.0	43	0.011	−3.30	−10.60%	1.07
	72.1 to 98.0	26	0.029	3.70	17.46%	1.17
Lateral gastrocnemius	−200.0 to −175.4	25	0.029	−3.43	−7.62%	1.05

*Note:* Values represent the characteristics of clusters where significant between‐group differences were detected (*p* < 0.05). Cluster location: The time interval (ms) relative to initial contact (0 ms) where the significant difference occurred. t*: The *t*‐value with the largest absolute magnitude within the cluster, retaining its sign. Positive values indicate non‐CAI > CAI, whereas negative values indicate CAI > non‐CAI. Mean _diff_: The average between‐group difference within the cluster (calculated as non‐CAI minus CAI). Cohen's *d* max: The maximum effect size within the cluster.

### 2.2 Walking Trapdoor

To simulate real‐world LASs during walking, a custom‐made walkway (0.8 *m* × 9.2 *m*) was employed (Figure 1). The substantial length of the walkway accommodated multiple gait cycles, ensuring participants achieved steady‐state gait and established a natural walking rhythm prior to perturbation onset. The walkway featured a built‐in trapdoor located at its midpoint. The tilting mechanism was secured by three removable locking pins at the base; upon disengagement, the platform of the trapdoor was mechanically released to undergo a rapid 24° inversion in the frontal plane [17]. Two reflective markers were placed on the lateral edge of the trapdoor platform to detect the onset of platform collapse. A perturbation magnitude of 24° was selected to align with established biomechanical safety thresholds, particularly for individuals with CAI, given that injury typically occurs when ankle inversion exceeds 30° [18].

**FIGURE 1:**
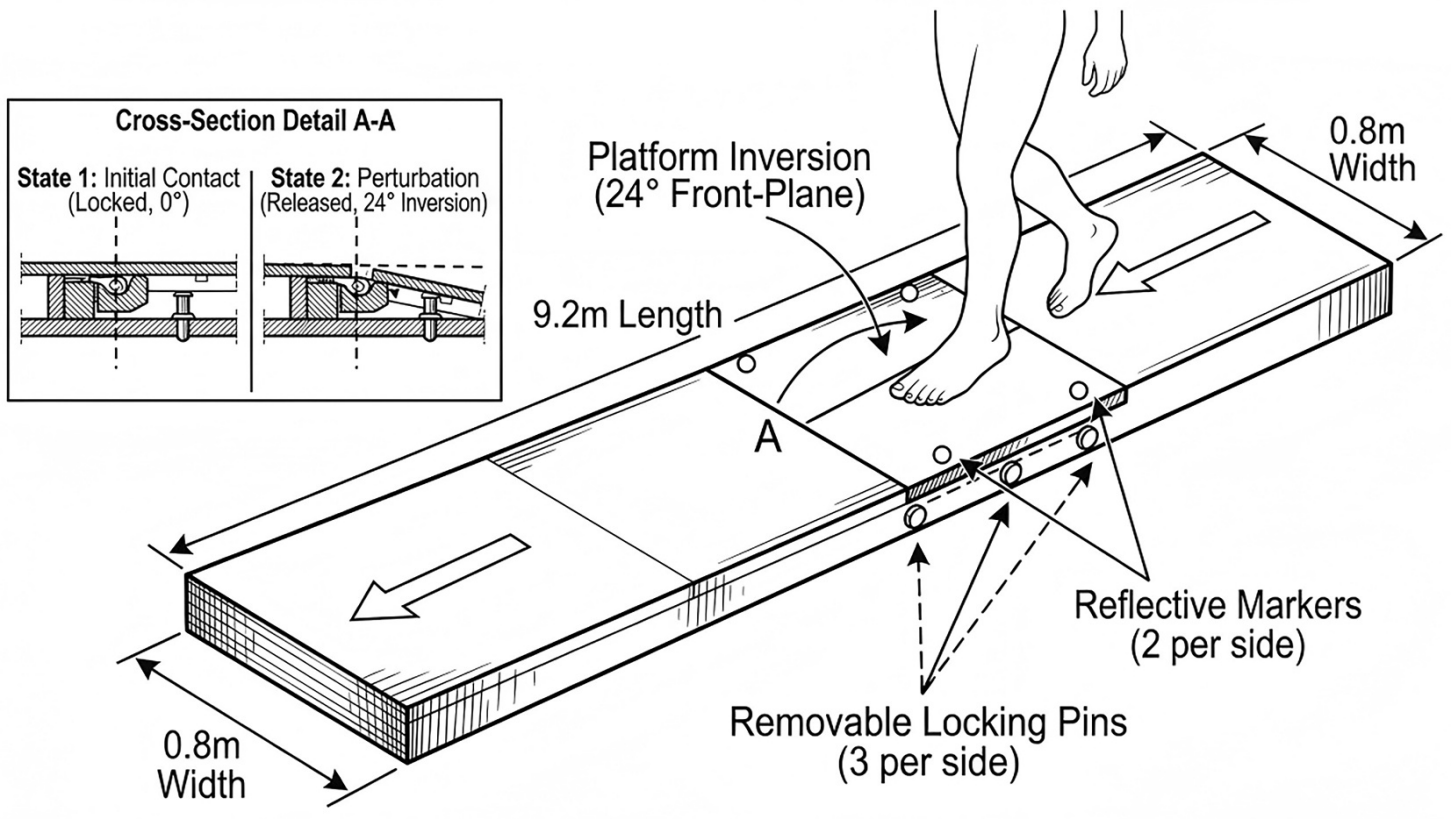
Walking trapdoor. The walkway (9.2 *m* length × 0.8 *m* width) was designed to accommodate multiple gait cycles, ensuring steady‐state walking prior to perturbation. The trapdoor mechanism is secured by three removable locking pins. Upon disengagement, the platform undergoes a rapid 24° inversion in the frontal plane to simulate a lateral ankle sprain. The enlarged panel illustrates the local mechanical structure of the trapdoor. Two reflective markers placed on the lateral edge of the platform were used to detect the onset of perturbation. Arrows indicate the direction of walking.

### 2.3 Protocols

Data were collected at the Biomechanics Laboratory of the University. Following informed consent and CAIT questionnaire completion, participants wearing standardized tight‐fitting clothing and footwear performed familiarization trials with the perturbation mechanism deactivated to establish a consistent starting position and ensure accurate foot placement without gait alterations. The unanticipated testing protocol comprised 15 walking trials, including at least 5 target inversion perturbations and 10 ‘dummy’ trials (locked trapdoor). To minimize habituation and anticipation bias, participants received standardized instructions prior to testing. They were informed that an ankle inversion perturbation might occur during walking trials, but remained naive to its presence, timing, or side. Before each trial, participants were verbally instructed to walk naturally at a comfortable, self‐selected pace while maintaining forward gaze on an eye‐level target fixed on the far wall. Environmental cues were neutralized using noise‐canceling headphones. Trials were presented in a standardized, blinded sequence, ensuring at least two dummy trials between each perturbation. To validate the unanticipated state, participants reported their pre‐trial expectations immediately after each walk. A trial was deemed a valid unanticipated event only when a predicted stable surface was followed by an actual drop and the participant maintained a continuous, natural gait without adjustments. For each participant, three valid trials were recorded and included in the analysis; these trials were selected chronologically as the first three perturbation trials that met the validity criteria. Any correctly anticipated perturbations were discarded and repeated. Participant safety was maintained via a ceiling‐mounted harness and a spotter. A 1‐min rest period was implemented between consecutive trials to minimize fatigue effects.

Ankle kinematics were recorded using a 12‐camera motion capture system (Vicon Motion Systems Ltd., Oxford, UK) at 200 Hz. The test limb was designated as the previously injured limb for individuals with CAI, and the preferred kicking limb for controls [19]. Forty‐two retro‐reflective markers were affixed to body anatomical landmarks in accordance with the Oxford Foot Model. To ensure consistency and minimize inter‐rater variability, a single trained researcher performed all marker placements and anthropometric measurements.

Muscle activity was recorded using a wireless surface EMG system (Noraxon, Scottsdale, AZ, USA) at 2000 Hz. Bipolar surface electrodes were positioned over the muscle bellies of the peroneus longus (PL), lateral gastrocnemius (LG), tibialis anterior (TA), and soleus (SOL), with an inter‐electrode distance of less than 2 cm. Prior to electrode application, standard skin preparation, including shaving and cleansing with 70% isopropyl alcohol, was conducted to minimize skin impedance and optimize signal fidelity.

### 2.4 Data Reduction

Kinematic and EMG data were analyzed within a 400‐ms window divided into two phases: the pre‐perturbation phase (200 ms prior to initial contact) and the post‐perturbation phase (200 ms following initial contact). In this study, initial contact was operationally defined as the onset of perturbation, identified by the initial vertical displacement of the marker on the platform's lateral edge. This time‐window was selected to capture the transition from preparatory activation to reactive stabilization, given that lateral ankle sprains typically occur within the first 200 ms post‐perturbation [18]. Time‐series data were normalized to 401 data points to facilitate Statistical Parametric Mapping (SPM) analysis of the entire waveform [20]. To determine whether the observed muscle activation patterns were unique to the perturbation event or reflected generalized gait adaptations, baseline unperturbed level walking strides from the same testing session were also analyzed. In accordance with standard gait analysis protocols [21], the unperturbed gait cycle was defined as the interval between two consecutive initial contacts (0%–100%) and time‐normalized to 101 data points.

Kinematic data were filtered using a fourth‐order zero‐lag Butterworth low‐pass filter with a cut‐off frequency of 10 Hz in Visual3D software (v6 Professional, C‐Motion, Germantown, MD, USA) [22]. Three‐dimensional ankle angles were calculated using an X‐Y‐Z Cardan rotation sequence, describing the orientation of the distal segment relative to the proximal segment's local coordinate system [23].

Raw EMG signals were processed using Noraxon MR3 software (Noraxon, Scottsdale, AZ, USA). Signals were band‐pass filtered (10–500 Hz) to attenuate noise and smoothed using a 50‐ms moving root‐mean‐square (RMS) window. The RMS envelopes were normalized to the task‐specific peak activation (i.e., the highest peak RMS observed across all valid trials) [24]. Task‐specific peak normalization was utilized over maximal voluntary isometric contraction (MVIC) to directly evaluate task‐evoked reactive patterns. This method avoids common MVIC confounders in this population, specifically AMI [25], pain‐limited effort [26], and the unsuitability of static references for rapid dynamic perturbations [27]. The processed, normalized waveforms were exported to MATLAB (MathWorks, Natick, MA, USA) for feature extraction.

### 2.5 Statistics

Independent‐sample t‐tests using Statistical Parametric Mapping (SPM) were performed to compare continuous ankle kinematics and muscle EMG between CAI and non‐CAI participants. Waveform analyses utilized the spm1d package (v0.4) in MATLAB (The MathWorks, Natick, MA, USA), with significance thresholds set by Random Field Theory. Discrete variables were analyzed using SPSS (Version 26.0, IBM Corp., Armonk, NY, USA). The Shapiro‐Wilk tests were used to assess data normality. Effect sizes were quantified using Cohen's *d*, with values interpreted as trivial (*d* < 0.20), small (*d* = 0.21–0.50), medium (*d* = 0.51–0.80), and large (*d* > 0.81). Statistical significance was set at *p* < 0.05.

## 3 Results

Walking speed did not significantly differ between groups (CAI: 1.39 ± 0.11 m/s; non‐CAI:1.43 ± 0.09 m/s; *p* = 0.223). As illustrated in Figure 2, no significant between‐group differences in ankle plantarflexion/dorsiflexion angles were detected (*p* > 0.05). Individuals with CAI exhibited significantly greater ankle inversion angles in four clusters, than those without CAI. Following perturbation, a cluster was identified at 0 to 111.0 ms (*p* < 0.001), followed by three clusters at 117.0 to 128.0 ms (*p* = 0.020), 142.0 to 145.0 ms (*p* = 0.047), and 154.0 to 165.0 ms (*p* = 0.019).

**FIGURE 2:**
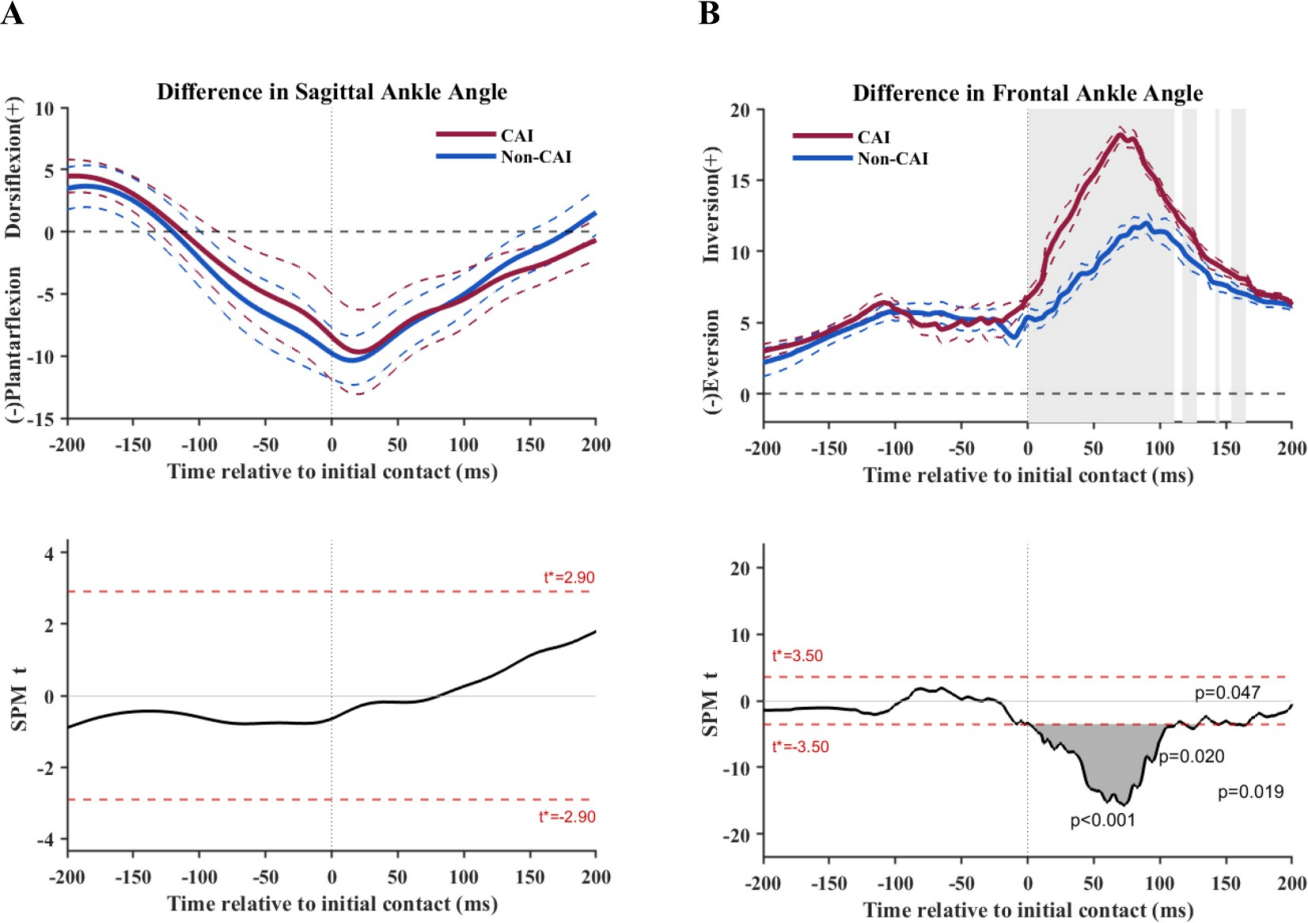
Ankle kinematics. (A) Ankle plantarflexion/dorsiflexion angles and (B) Ankle inversion/eversion angles. Upper panels: Ankle joint angle waveforms (solid lines) bounded by 95% confidence intervals (shaded bands) for the CAI (red) and non‐CAI (blue) participants. Lower panels: SPM{*t*} trajectories derived from independent sample t‐tests. The horizontal red dashed lines indicate the critical random field threshold (t*). Grey shaded regions denote clusters where the SPM{*t*} curve exceeds t*, indicating statistically significant between‐group differences (*p* < 0.05).

As illustrated in Figure 3, no significant between‐group differences were detected in activation of the TA and SOL (*p* > 0.05). Prior to perturbation, individuals with CAI demonstrated significantly greater PL pre‐activation in two clusters (−200.0 to −196.4 ms, *p* = 0.049; −54.6 to −11.0 ms, *p* = 0.011). Conversely, post‐perturbation, individuals with CAI showed significantly lower PL activation (72.1 to 98.0 ms, *p* = 0.029). Additionally, individuals with CAI displayed significantly greater LG pre‐activation than those without CAI (−200.0 to −175.4 ms, *p* = 0.029) prior to perturbation. Details can be seen in Table 1.

**FIGURE 3:**
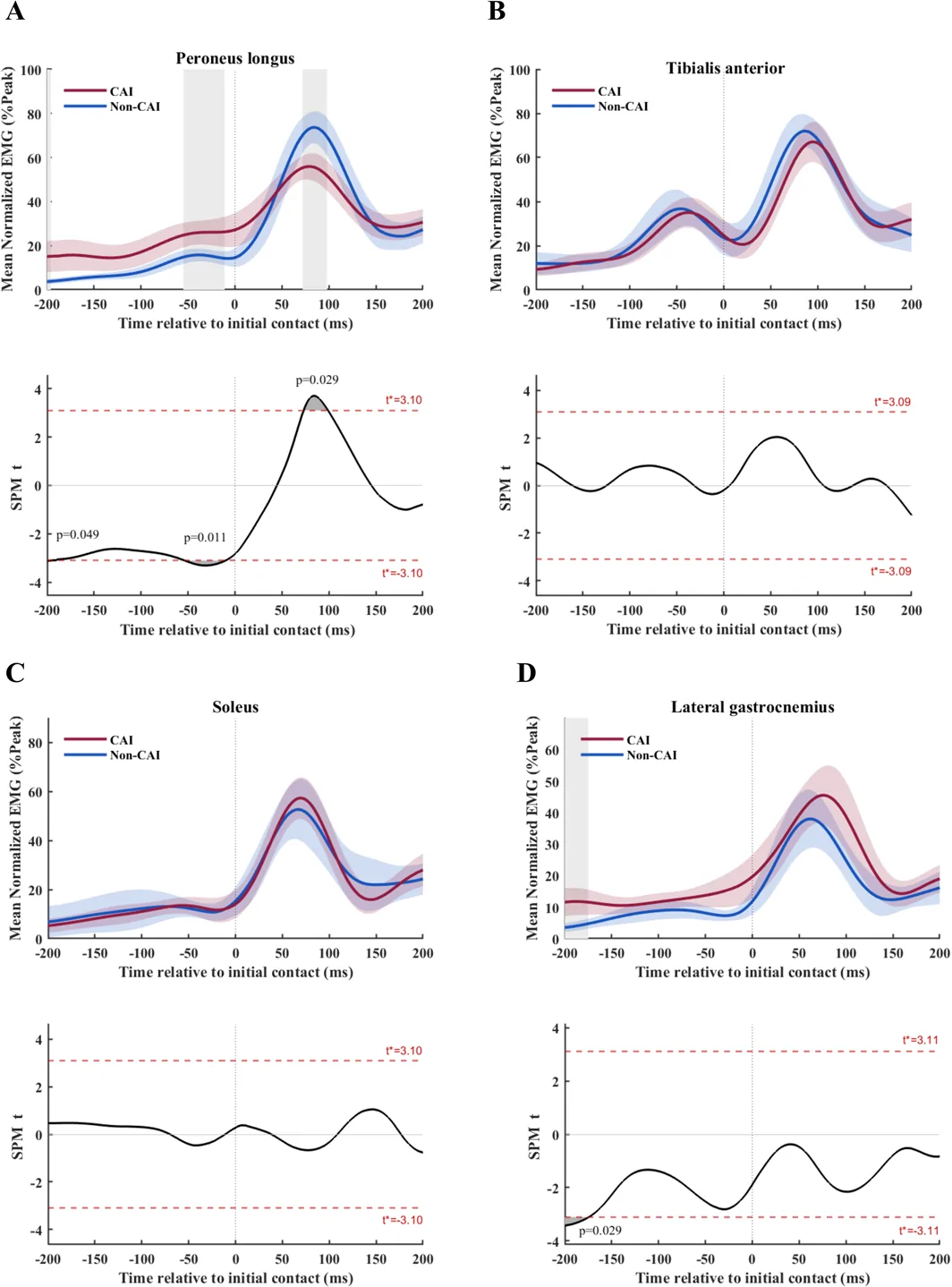
Muscle activation. (A) Peroneus Longus (B) Tibialis Anterior (C) Soleus, and (D) Lateral Gastrocnemius. Upper panels: Mean normalized EMG waveforms (solid lines) with 95% confidence intervals (shaded bands) for CAI (red) and non‐CAI (blue) participants. Lower panels: SPM{*t*} trajectories derived from independent sample t‐tests. The horizontal red dashed lines indicate the critical random field threshold (t*). Grey shaded regions denote supra‐threshold clusters where the SPM{*t*} curve exceeds t*, indicating statistically significant between‐group differences (*p* < 0.05).

During unperturbed level walking, SPM analysis revealed that individuals with CAI exhibited significantly higher PL activation during the terminal swing phase (86.9%–95.0% of the gait cycle, *p* = 0.007) immediately prior to initial contact compared with those without CAI (Figure 4).

**FIGURE 4:**
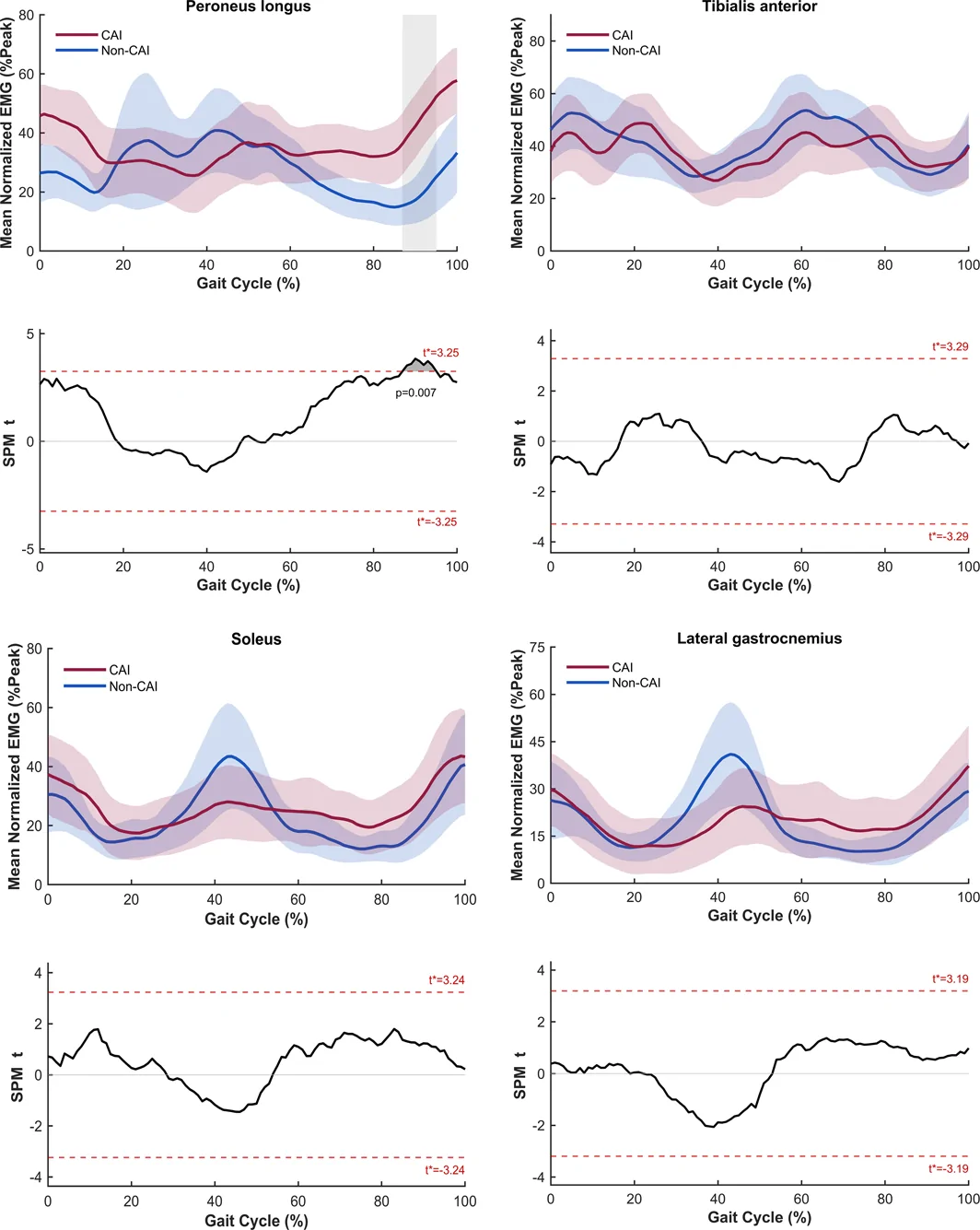
Muscle activation (level walking). (A) Peroneus Longus (B) Tibialis Anterior (C) Soleus, and (D) Lateral Gastrocnemius. Upper panels: Mean normalized EMG waveforms (solid lines) with 95% confidence intervals (shaded bands) for CAI (red) and non‐CAI (blue) participants. Lower panels: SPM{*t*} trajectories derived from independent sample t‐tests. The horizontal red dashed lines indicate the critical random field threshold (t*). Grey shaded regions denote supra‐threshold clusters where the SPM{*t*} curve exceeds t*, indicating statistically significant between‐group differences (*p* < 0.05).

## 4 Discussion

Building on previous ankle inversion perturbation models, the present study extends previous work by examining continuous ankle kinematics and lower‐leg muscle activation before and after unanticipated inversion perturbations during walking in individuals with and without CAI. The results supported Hypothesis #1, which predicted that individuals with CAI would exhibit higher feedforward pre‐activation in lateral ankle muscles. Our data also supported Hypothesis #2, suggesting that feedforward pre‐activation may effectively counteract ankle inversion tendencies prior to perturbation. Conversely, Hypothesis #3 was rejected, inferring that this feedforward pre‐activation was insufficient to prevent excessive ankle inversion post‐perturbation.

Our EMG data revealed that individuals with CAI exhibited higher pre‐activation of the lateral ankle muscles than their counterparts prior to perturbation. This observation is consistent with prior research conducted during level walking scenarios [11, 28], yet it diverges from findings reported during drop‐landing scenarios [9]. We attribute this discrepancy to task dependency. Unlike landing, the stereotyped walking gait provides the CNS with a sufficient temporal window to modulate movement strategies. From a functional perspective, given that physiological latencies often render reflexive muscle activation too slow for immediate protection [7], this enhanced pre‐activation functions as a feedforward compensatory mechanism [8]. Crucially, our baseline gait analysis revealed that elevated PL pre‐activation is not merely a task‐specific response to the threat of perturbation, but rather a generalized, habitual motor adaptation present during unperturbed level walking. Specifically, individuals with CAI exhibited significantly higher PL activation during the terminal swing phase immediately before foot contact. This baseline elevation suggests that, in response to the passive instability caused by lateral ligament laxity, individuals with CAI appear to adopt a ‘protective stiffening’ or ‘fear‐avoidance’strategy [28]. Higher pre‐activation of lateral ankle muscles is likely driven by the chronic deafferentation of mechanoreceptors in the lateral ligaments, which prompts the CNS to increase gamma‐motoneuron drive prior to perturbation [29]. By augmenting alpha‐gamma co‐activation, the CNS attempts to heighten the muscle spindles' sensitivity to stretch, thereby compensating for the compromised ligamentous feedback [30]. However, as these specific neurophysiological and psychological parameters were not directly measured in the present study, these mechanisms serve as plausible theoretical frameworks rather than confirmed findings, warranting future targeted investigations.

Our kinematic data showed no significant differences in ankle inversion between individuals with and without CAI prior to perturbation, consistent with previous gait studies [31]. This maintenance of normal alignment occurs despite the fact that individuals with CAI may suffer from ligamentous laxity [32]. Our data suggest that the CNS overcomes these impairments via higher feedforward activation. Specifically, the increased pre‐activation of lateral ankle muscles enhances ankle stiffness, which counters the tendency for ankle inversion [22]. This active mechanism effectively offsets the inadequate joint constraint potentially resulting from lateral ligament laxity, thereby minimizing ankle inversion in individuals with CAI prior to perturbation.

Our kinematic data further demonstrate that, post‐perturbation, individuals with CAI exhibit significantly greater ankle inversion compared with those without CAI. This finding advances the existing literature by demonstrating that the habitual feedforward pre‐activation observed in individuals with CAI is functionally insufficient to prevent excessive joint kinematics during unexpected dynamic inversions. Consistent with the conclusions of Feger et al. [4], this implies that although elevated pre‐activation serves as an adequate compensatory strategy for maintaining steady‐state gait, it fails to generate the requisite resistive torque to counteract the high‐velocity demands of dynamic external perturbations. This inadequacy may be attributed to the potential pathological ligament laxity in CAI patients, which necessitates further shortening of the lateral ankle muscles to ‘take up the slack’ [33], thereby rendering the pre‐activation insufficient under sudden inversion conditions. This leads to a vulnerable foot posture [34] and a peak inversion angle of approximately 16° that surpasses the critical injury threshold of 15° [35], consequently exposing individuals with CAI to an ongoing risk of recurrent injury.

Our findings further reveal that, during the post‐perturbation phase, individuals with CAI showed lower PL activation compared with those without CAI. This could be considered a secondary factor contributing to greater ankle inversion in CAI participants, as diminished PL activation is linked to larger inversion angles and an increased risk of recurrent sprains [10]. This finding contrasts with Simpson et al. [9], who reported no significant differences; this discrepancy is likely attributable to methodological choices. Previous studies often relied on discrete scalar values (e.g., average EMG amplitudes) that may mask transient fluctuations [9]; conversely, our use of SPM to analyze the entire waveform provided greater sensitivity to capture these neuromuscular deficits [36]. Crucially, the identified PL activation deficit at 72.1–98.0 ms falls within the medium‐to long‐latency response (MLR/LLR) windows [37, 38]. This reactive failure points to an impaired eversion defense driven by two interconnected neuropathological mechanisms. First, structural damage to lateral passive elements (e.g., ligaments and joint capsule) disrupts mechanoreceptor afferents, compromising central sensorimotor integration of ankle position and kinematics [39]. Second, one plausible explanation for the reduced post‐perturbation activation is arthrogenic muscle inhibition (AMI). Persistent abnormal afferent input has been proposed to contribute to AMI by reducing gamma‐motoneuron excitability and muscle spindle sensitivity [25, 40]. Because MLR and LLR components involve polysynaptic spinal and supraspinal sensorimotor pathways [41], the observed activation deficit may reflect altered task‐specific reflex regulation rather than a simple peripheral delay. Nevertheless, because these spinal and supraspinal pathways were not directly assessed, AMI should be regarded as a plausible but unconfirmed explanation for the observed activation deficit.

From a clinical translation perspective, traditional muscle strengthening [42] and balance training [43] remain important components of CAI rehabilitation. However, our findings suggest that restoring baseline strength and postural control alone may not fully address the neuromuscular deficits that emerge during unexpected dynamic perturbations. Specifically, individuals with CAI exhibited elevated pre‐activation before perturbation; however, this response was insufficient to prevent excessive inversion and was followed by reduced PL activation during the early post‐perturbation phase. These findings highlight the need to complement conventional rehabilitation with interventions targeting rapid reactive neuromuscular control. Unexpected perturbation training may be particularly relevant because it requires the lateral ankle muscles to generate rapid feedback responses to sudden joint displacement, potentially improving the ability to control unexpected inversion and reduce recurrent sprain risk.

This study has several limitations. First, while rigorous methodological controls (e.g., randomization, dummy trials, sensory masking) minimized explicit anticipation, such expectancy cannot be completely eliminated in repeated laboratory perturbation paradigms and may limit the generalizability of the findings to truly unexpected real‐world ankle sprains. Second, the participants consisted primarily of young adults, limiting the generalizability of our findings to other age populations. Finally, this study did not include a ‘coper’ group. Without this comparison, it remains unclear whether the heightened feedforward pre‐activation is a maladaptive trait specific to CAI or a general adaptive response following ankle injury. Future studies should incorporate copers to clarify these sensorimotor adaptation mechanisms.

## 5 Conclusion

Individuals with CAI demonstrated higher feedforward pre‐activation in the PL and LG. Although the pre‐activation may keep ankle inversion during stereotyped walking, it may be inadequate to counteract excessive ankle inversion following a sudden trapdoor‐induced landing.

## Author Contributions

**Wenqi Ran:** methodology, investigation, data curation, formal analysis, visualization, writing – original draft. **Qipeng Song:** conceptualization, validation, writing – review and editing. **Wei Yin:** investigation, software, supervision.

## Funding

The authors have nothing to report.

## Ethics Statement

The study was approved by the Ethics Committee of ShanDong Sport University (Approval No. 2025053) and conducted in accordance with the Declaration of Helsinki. All participants provided written informed consent prior to participation.

## Conflicts of Interest

The authors declare no conflicts of interest.

## Data Availability

The data that support the findings of this study are available on request from the corresponding author. The data are not publicly available due to privacy or ethical restrictions.
